# ﻿The ecology of lichenicolous lichens: a case-study in Italy

**DOI:** 10.3897/mycokeys.105.121001

**Published:** 2024-05-31

**Authors:** Pier Luigi Nimis, Elena Pittao, Monica Caramia, Piero Pitacco, Stefano Martellos, Lucia Muggia

**Affiliations:** 1 University of Trieste, Department of Life Sciences, via Giorgieri 10, 34127 Trieste, Italy University of Trieste Trieste Italy

**Keywords:** Algal theft, host, lichenised fungi, photobiont, sexual reproduction, symbioses

## Abstract

This paper, with Italy as a case-study, provides a general overview on the ecology of lichenicolous lichens, i.e. those which start their life-cycle on the thallus of other lichens. It aims at testing whether some ecological factors do exert a positive selective pressure on the lichenicolous lifestyle. The incidence of some biological traits (photobionts, growth-forms and reproductive strategies) in lichenicolous and non-lichenicolous lichens was compared, on a set of 3005 infrageneric taxa potentially occurring in Italy, 189 of which are lichenicolous. Lichenicolous lichens have a much higher incidence of coccoid (non-trentepohlioid) green algae, crustose growth-forms and sexual reproduction. A matrix of the 2762 species with phycobionts and some main ecological descriptors was subjected to ordination. Lichenicolous lichens occupy a well-defined portion of the ecological space, tending to grow on rocks in dry, well-lit habitats where a germinating spore is likely to have a short life-span, at all altitudes. This corroborates the hypothesis that at least some of them are not true “parasites”, as they are often called, but gather the photobionts - which have already adapted to local ecological conditions - from their hosts, eventually developing an independent thallus.

## ﻿Introduction

Lichens are a symbiosis between a fungal partner, the mycobiont and one or more photosynthetic partners, the photobionts, which is either a cyanobacterium (cyanobiont), a green microalga (phycobiont) or both (Hawksworth 1988; Spribille et al. 2022; Sanders 2023, 2024). The photobiont is a carbon source for the heterotrophic mycobiont (Nash 2008) and a nitrogen source for cyanolichens, due to the cyanobacterium fixing the atmospheric nitrogen (Rikkinen 2002). In return, the mycobiont provides the photobiont with optimal living conditions, protecting it from high temperatures, light (UV radiation) and drought (Palmqvist et al. 2008; Grube 2018). Some authors regard lichens as an example of controlled-parasitism, since the fungus seems to obtain most of the benefits from the photobionts and to control them (Richardson 1999; Nash 2008). Many other organisms have been found dwelling on the surface of or within lichen thalli (Honegger 1992; Bates et al. 2011), such as non-photosynthetic bacteria (Grube 2018), unicellular basidiomycete yeasts (Spribille et al. 2016) and non-lichenised fungi (Hawksworth 1988; Arnold et al. 2009; Muggia et al. 2016; Diederich et al. 2018). Thus, lichens were recently re-defined as self-sustaining microecosystems (Insarova and Blagoveshchenskaya 2016; Hawksworth and Grube 2020, but see also the criticism by Sanders 2024)). Additional complexity was reported inside a single lichen thallus by the co-existence of multiple phycobionts (del Campo et al. 2012; Muggia et al. 2014; Moya et al. 2017; Moya et al. 2020) which respond differently to abiotic stressors and perhaps also of multiple mycobionts (Ament-Velásquez et al. 2021). Phycobiont co-existence is advantageous for lichens under extreme environmental conditions, in which this phenomenon seems to be common (del Hoyo et al. 2011; Casano et al. 2011, 2015). Lichens also host many lichenicolous, non-lichenised fungi which gain their nutrition from the host lichen thallus, draining it of its photosynthetic products, thus being regarded as parasitic or saprophytic (Hawksworth 1988; Rambold and Triebel 1992; Hafellner 2018) going as far as being necrotrophic when they have devastating effects on either the mycobiont (Diederich 1996; de los Rìos and Grube 2000) or the photobiont (Grube and Hafellner 1990).

A peculiar case is that of lichenicolous lichens, which regularly start their life-cycle on the thalli of other lichen species, eventually building their own lichenised thallus (Poelt 1958, 1990; Rambold and Triebel 1992, Diederich et al. 2018). Some of them are specialists, i.e. they can only grow on a certain species of lichen, others are more generalists (Moya et al. 2020). Some lichenicolous lichens simply overgrow other lichens in ecological successions because of space competition (Armstrong and Welch 2007). Others, the so-called cyanotrophic lichens, are green algal lichens that grow on free-living cyanobacteria or cyanobacterial lichens, probably to benefit from their nitrogen-fixing capability (Poelt and Mayrhofer 1988; Rikkinen 2002; Honegger 2012a). Finally, others always start the life-cycle on lichens with the same general type of photobiont. The latter, which are the object of the present study, are often referred to as “parasites” (Poelt 1958; Honegger 2012b), although according to several authors (e.g., Richardson (1999); Diederich et al. (2018); Moya et al. (2020)), they take over the photobiont from the host to avoid re-establishing the symbiosis by searching for a new photobiont of their own. Once the photobiont has been acquired, it can be maintained or be substituted with a different and often more favourable algal partner through algal switching (Friedl 1987; Piercey-Normore and De Priest 2001; Moya et al. 2020). To our knowledge, no large-scale assessment of species traits and ecology of the total lichenicolous lichen biota across a broad spectrum of ecological conditions was ever attempted. Taking advantage of the availability of ecological indicator values for all lichens of Italy (Nimis 2016), we have tried to provide such an overview at the level of a well-known, rich lichen flora encompassing several biomes, as that of Italy. The main aim of this paper is to test whether lichenicolous lichens differ from non-lichenicolous lichens in their ecology, i.e. whether some ecological factors could be detected, which may exert a positive selective pressure on the acquisition of a lichenicolous life-style.

## ﻿Material and methods

The list of lichenicolous and non-lichenicolous lichens, their bio-morphological traits and their ecological descriptors were retrieved from Nimis and Martellos (2023). We have considered all lichen species reported from Italy, plus those known from neighbouring countries, whose presence in Italy is possible.

The bio-morphological traits are:

1. ***Photobionts* : Ch** (phycobiont: green algae other than *Trentepohlia*), **Tr** (phycobiont: *Trentepohlia*), **Cy.h** (cyanobiont, filamentous), **Cy.c** (cyanobiont, coccoid);
2. ***Reproductive strategies* : A.f** (mainly asexual, by thallus fragmentation), **A.i** (mainly asexual, by isidia or isidia-like structures), **A.s** (mainly asexual, by soredia or soredia-like structures), **S** (mainly sexual, meiotic spores of the mycobiont);
3. ***Growth forms* : Cr** (crustose), **Fol** (foliose), **Frut** (fruticose), **Lepr** (leprose), **Sq** (squamulose).

The ecological descriptors are:

1. ***Substrata* : Epiph** (epiphytic: on bark, leaves, lignum), **Sax** (saxicolous: on rocks), **Terr** (terricolous: on soil, terricolous mosses and plant debris);
2. ***Phytoclimatic range* : Oc** (oceanic: restricted to areas with a humid-warm oceanic climate), **Suboc** (suboceanic: most common in areas with a humid-warm climate), **Subc** (subcontinental: restricted to areas with a dry-subcontinental climate);
3. ***Altitudinal distribution (vegetation belts, as a proxy of temperature)* : A1** (eu-Mediterranean), **A2** (submediterranean), **A3** (montane), **A4** (subalpine and oroboreal), **A5** (alpine), **A6** (nival);
4. ***Poleotolerance (tolerance to anthropization)*** : from **Pol3** (species occurring in heavily disturbed areas) to **Pol0** (species exclusively occurring on old trees in ancient, undisturbed forests);
5. ***Ecological indicator values*** : these are “expert assessments” that qualitatively express the ecological range of species with respect to different factors on a 5-class ordinal scale (see Nimis (2016)). The predictivity of the values used in this study was tested against real data (Nimis and Martellos 2001) and proved to be high.

- ***pH of the substratum*** : from **pH1** (very acid substrata) to **pH5** (basic substrata);
- ***Light (solar irradiation)*** : from **L1** (in very shaded situations) to **L5** (in sites with high direct solar irradiation);
- ***Xerophytism (aridity)*** : from **X1** (hydro- and hygrophytic, in aquatic or marine situations or sites with a very high frequency of fog) to **X5** (very xerophytic);
- ***Eutrophication*** : from **E1** (not resistant to eutrophication) to **E5** (occurring in highly eutrophicated situations).

Data analysis was performed with the R 4.3.0 software (R Core Team 2023). Differences between lichenicolous and non-lichenicolous lichens were tested separately for growth forms, photobionts and reproductive strategies using Pearson’s Chi-squared test in the package Rcmdr (Fox and Bouchet-Valat 2023). In order to test whether lichenicolous lichens occupy a well-delimited portion of the ecological space, as compared with non-lichenicolous lichens, the presence-absence matrix of species and ecological descriptors was subjected to Non-metric Multidimensional Scaling (NMDS) ordination after loading the vegan package (Oksanen et al. 2022). The function metaMDS, with Jaccard as dissimilarity index was used. The statistical significance of differences in ecological space occupancy was also tested on the same dissimilarity matrix used for NMDS, with an analysis of multivariate homogeneity of groups dispersions (function BetaDispersion 2.0, Bacaro et al. (2012, 2013)) and a Permutational Multivariate Analysis of Variance (function adonis2). Due to the absence of lichenicolous lichens with cyanobacteria as the main photobiont (see Results), cyanolichens were excluded from this analysis.

## ﻿Results

On a total of 3005 lichenised species potentially occurring in Italy, 189 were retained as “lichenicolous”. The mycobionts of the latter are phylogenetically clustered, most of the species in our dataset belonging to the *Lecanoromycetes* (84.4%), followed by the *Eurotiomycetes* (14.5%). The same applies for their hosts, which mostly belong to the *Lecanoromycetes* (95.3%), followed by the *Eurotiomycetes* (4%).

Table 1 compares the bio-morphological traits of lichenicolous and non-lichenicolous taxa. Lichenicolous lichens significantly differ from the other lichen species in growth forms, photobionts and reproductive strategies (Pearson’s Chi-squared test, p < 0.001) and show the highest incidence of crustose forms reproducing sexually, most of them with a green, non-trentepohlioid photobiont.

**Table 1.** Comparison of some main biological traits between lichenicolous and non-lichenicolous lichens potentially occurring in Italy (3005 species). All differences are highly significant (p < 0.001).

**Bio-morphological traits**	**Lichenicolous**		**Non-lichenicolous**	
	**189 taxa**		**2816 taxa**	
	**n**	%	**n**	%
**Crustose**	182	96	2041	72
**Foliose**	0	0	358	13
**Fruticose**	0	0	244	9
**Leprose**	0	0	32	1
**Squamulose**	7	4	141	5
**Cyanobacteria coccaceous**	1	1	52	2
**Cyanobacteria filamentous**	0	0	190	7
**Green algae(excl. *Trentepohlia*)**	186	98	2322	82
***Trentepohlia***	2	1	252	9
**Asexual (fragmentation)**	0	0	39	1
**Asexual (isidia)**	4	2	113	4
**Asexual (soredia)**	5	3	480	17
**Asexual (other)**	1	1	5	0
**Sexual**	179	95	2184	78

**Figure 1.**
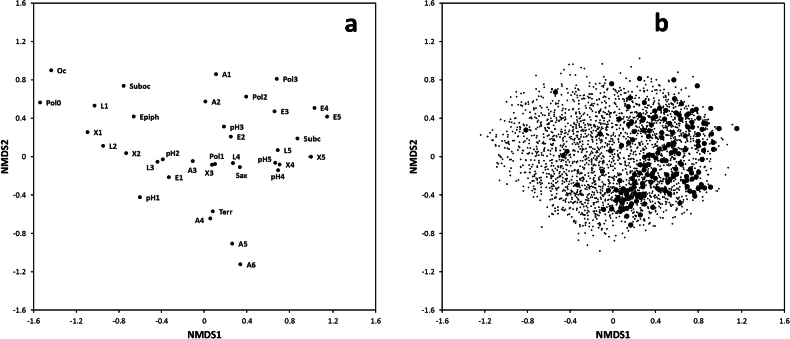
NMDS ordination of ecological descriptors (**a**) and of the 2762 species of phycolichens potentially occurring in Italy, with lichenicolous taxa flagged by larger dots (**b**). For abbreviations, see Material and methods.

Fig. 1 shows the NMDS ordination (stress value 0.226) of ecological descriptors (a) and species (b), limited to the 2762 phycolichens. In Fig. 1a, the first axis, from negative to positive scores, reflects a gradient of increasing aridity and solar irradiation, with epiphytic species tending to have negative scores, saxicolous species positive scores and terricolous species occupying an intermediate position. The second axis reflects a gradient, from positive to negative scores, of increasing altitude/decreasing temperatures. Thus, the two axes in Fig. 1a describe an ecological space mainly defined by water (first axis) and temperature (second axis). Tolerance to eutrophication is most frequent amongst species growing in dry sites at low elevations, i.e. where human influence (agriculture, urbanisation) is the highest. The pH of the substrate seems to be less relevant, with a tendency for species growing on basic substrata to be most frequent on rocks in arid and well-lit situations, probably due to the prevalence of calcareous substrata throughout the country. Oceanic and suboceanic species tend to be bound, as it could be expected, to undisturbed, low-elevation, humid-shaded situations, for example, in lowland forests, while subcontinental species appear to be mostly saxicolous in dry situations. Lichenicolous lichens significantly differ (p < 0.001) from the other lichen species in ecological space occupation. Fig. 1b shows the occupancy of the ecological space depicted in Fig. 1a by phycolichens: lichenicolous taxa clearly tend to occupy a well-defined portion of the ecological space, i.e. to have positive scores on the first axis. Table 2 shows the distribution of the values of ecological descriptors in lichenicolous and non-lichenicolous phycolichens. Lichenicolous species differ from non-lichenicolous species in the higher percentage of saxicolous species and the higher values of the xerophytism index, followed by that, partly related, of solar irradiation, while the incidence of oceanic and suboceanic species is lower and that of subcontinental species is higher. Altitude/temperature, eutrophication, pH and poleophoby do not differentiate between the two groups.

**Table 2.** Distribution of the values of ecological descriptors in lichenicolous and non-lichenicolous phycolichens.

Ecological descriptors	Lichenicolous		Non-lichenicolous	
	(188 taxa)		(2574 taxa)	
	n	%	n	%
**Epiph**	7	4	978	38
**Sax**	173	92	1394	54
**Terr**	15	8	460	18
**Oc**	0	0	48	2
**Suboc**	9	5	434	17
**Subc**	17	9	84	3
**A1**	75	40	955	37
**A2**	82	44	1248	48
**A3**	101	54	1590	62
**A4**	113	60	1377	53
**A5**	106	56	879	34
**A6**	10	5	137	5
**Pol3**	3	2	99	4
**Pol2**	23	12	514	20
**Pol1**	186	99	2340	91
**Pol0**	1	1	212	8
**pH1**	50	27	969	38
**pH2**	106	56	1635	64
**pH3**	96	51	1281	50
**pH4**	65	35	747	29
**pH5**	56	30	548	21
**L1**	2	1	64	2
**L2**	6	3	476	18
**L3**	42	22	1577	61
**L4**	175	93	1927	75
**L5**	109	58	831	32
**X1**	4	2	359	14
**X2**	10	5	1169	45
**X3**	87	46	1529	59
**X4**	159	85	981	38
**X5**	128	68	225	9
**E1**	99	53	1902	74
**E2**	121	64	1260	49
**E3**	79	42	777	30
**E4**	38	20	284	11
**E5**	7	4	72	3

## ﻿Discussion

Lichenicolous lichens proved to be a biologically and ecologically very well-defined guild of species. Most of them reproduce sexually, have a crustose growth-form, a green, non-trentepohlioid photobiont and live on rocks in dry and very well-lit situations, at all altitudes.

Sexual reproduction requires the fungal hypha of the mycobiont to encounter a suitable photobiont to re-establish the symbiosis (Seymour et al. 2005). On the other hand, asexual reproduction consists of vegetative propagules, for example, isidia and soredia, which contain both the fungal and photosynthetic partner, being dispersed simultaneously and establishing a new thallus (Ott 1987). According to Poelt (1993), soredia are the smallest form of a miniaturised lichen and the most successful way to ensure co-dispersion of the two symbionts in a new site. The mycobiont is considered an obligate biont since it cannot occur free-living, because of its slow growth in isolation and incapability to compete with other fungi, while free-living photobionts may be common, especially in humid and moist terrestrial habitats (Nash 2008). One may, therefore, assume that asexual reproduction should be most common amongst lichens dwelling in dry and well-lit conditions, which may be unfavourable to a delicate germinating spore and perhaps also to free-living green algae. However, Nimis and Martellos (2003) have shown that sorediate lichens have a higher incidence in humid-shaded situations and are scarce both in dry, well-lit habitats and in periodically submerged sites, where sexual reproduction is prevalent. The very few lichenicolous lichens in our dataset which do not occur in dry sites – see Fig. 1b – are almost all hydrophytic species.

Both sexual and asexual reproduction have their disadvantages: sexual reproduction has a high metabolic cost and subjects the lichen to low biotic pressures in high-stress environments (Seymour et al. 2005); asexual reproduction implies low genetic recombination and, hence, a lower potential for evolutionary development (Nash 2008). Sexual reproduction could, thus, be essential to lichens of high-stress environments, providing enhanced genetic variability and a high chance of adaptation and survival. This implies also that the mycobiont is more flexible in creating a symbiosis with the better-adapted photobiont amongst those that are compatible. Lichenicolous mycobionts would take advantage of the algae available in the host thallus, thus avoiding the effort of finding an appropriate algal partner (Friedl 1987; Wedin et al. 2016; Moya et al. 2020) and, at the same time, being totally constrained by the photobionts associated with their host. One could object that in a highly stressful environment, such as city downtowns, species with vegetative propagules could prevail (see, for example, Gilbert (1990)). However, Nimis and Martellos (2003) showed that, at least in Italy, the prevalence of lichens with asexual reproduction in urban environments is overestimated, as it involves only very few (less than l% of the total), abundant and common species. In this case, asexual reproduction could be an advantageous propagation strategy of a few r-selected species which can be accommodated within the strategy group of stress-tolerant ruderals (see also Rogers (1990); Jahns and Ott (1997)).

The absolute prevalence of crustose, saxicolous life-forms in lichenicolous lichens may be related to their high frequency in dry situations. Crustose lichens are the slowest growing of all lichens, which allows them to have a lower demand for nutrients than foliose or fruticose lichens, therefore enabling colonisation of harsher environments (Armstrong and Bradwell 2010). They are also intimately associated with the substratum and their metabolic growth rate relies on its water holding capacity, which is generally much lower in rocks than in bark or soil (Green and Lange 1995).

The scarcity of trentepohlioid photobionts in lichenicolous lichens is probably due to the fact that *Trentepohlia*, a genus of filamentous green algae, is bound to shaded-humid and warm conditions, where it often occurs in the free state. Trentepohlioid lichens indeed have their maximum diversity in tropical evergreen rainforests, where solar irradiance is low and air humidity is high (Friedl and Büdel 2012; Matos et al. 2015; Martellos et al. 2020). Finally, the scarcity of lichenicolous cyanolichens may be due to a different reason. Cyanobacteria dominate many extreme, arid environments, reaching temperatures up to 73 °C, thanks to their tolerance of desiccation and water stress, being abundantly available in the free state for lichen symbiosis in dry sites (Pentecost and Whitton 2000; Whitton and Potts 2000; Nimis et al. 2020). It has long been known that very dry, steeply inclined rocks surfaces host visually conspicuous cyanobacterial films (“Tintenstriche”, Lüttge (1997)), with a very rich diversity in species (Pentecost and Whitton 2000; Nimis et al. 2020). Many mycobionts of cyanolichens may, therefore, not need to develop a lichenicolous lifestyle for acquiring their photobionts, as they would find ecologically adapted cyanobionts already available in the environment. There could be, however, an alternative reason for the scarcity of lichenicolous species in cyanolichens and phycolichens with *Trentepohlia*; the fact that fungi in lichenicolous lichens mostly belong to the Lecanoromycetes. The process of host colonisation could be related not only to the photobiont of the host, but also to certain mycobiont traits, such as biochemical defences to fungal invasion, likely having a relevant role in the distribution of lichenicolous fungi across the lichenised lineages of Ascomycota.

The ecological conditions prevailing on well-lit, dry rock surfaces with low water-holding capacity may be unfavourable for the establishment of lichens reproducing sexually. Once a spore falls in a suitable habitat it germinates, generating a delicate mycelium which eagerly looks for a compatible photosynthetic partner to re-build the lichen symbiosis before being destroyed by a hostile environment where water is scarce and temperatures may be high due to strong solar irradiation (Pyatt 1973; Ott 1987). It is not clear whether the possible scarcity of free-living algae in dry sites could also play a role in the acquisition of a lichenicolous life-style. For lichens of dry habitats, the probability for a germinating spore to find a suitable alga has been estimated to be extremely low by Scott (1971) and some authors (e.g. Guillitte (1993)) have found that free-living green algae are quite rare on dry rock surfaces. However, other authors (e.g. Yung et al. (2014)) have demonstrated the presence of algal species, able to lichenise, in dry environments where mycobiont species have not been recorded. In any case, an original solution to the difficulties in the lichenisation of sexually reproducing species in very dry sites, suggested by several authors, might be that of “stealing” the photobiont from the thalli of other species (Rambold and Triebel 1992; Richardson 1999), which would explain their lichenicolous lifestyle. Lichenicolous phycolichens are commonly referred to as “parasites” (Poelt and Doppelbaur 1956; Poelt 1958) and as such they are usually flagged in several modern floras and checklists (e.g. Clauzade and Roux (1985); Wirth et al. (2013); Nimis et al. (2018)). According to Smith and Smith (2015), a parasite is an organism which benefits from the tight and prolonged association with the host, which is progressively damaged and exploited to derive nourishment and a habitat. A parasite was also defined as an organism that lives on and at the expense of a host, implying a metabolic dependence from it (Esch and Fernandez 1993). Considering these definitions, the term “parasite” may not be appropriate for many lichenicolous lichens, since, at least in later life-stages, they derive nutrients from their own photobionts and not from the lichen host, as instead the lichenicolous, non-lichenised fungi do. The prolonged persistence upon the host was considered a characteristic of a parasite by Poelt and Doppelbaur (1956). While some lichenicolous lichens may be confined to the host thalli throughout their lifetime, others can become independent, not using the host as a lifelong habitat (Richardson 1999; Honegger 2012b; Moya et al. 2020). Moreover, the degree of colonisation and, thus, of damage to the host, also varies, as its thallus can be either locally or completely overgrown and replaced (Richardson 1999; Honegger 2012b). Hence, since the range of interactions is broad and the transitions fluid, the term “parasite” for lichenicolous phycolichens should be best reserved for those producing clear damage or even the disappearance of the host thallus.

The concept of “stealing of the phycobiont”, though, should also be re-considered. Indeed, the lichenicolous mycobiont does not depredate the lichen host from its photosynthetic partner, but it takes some of the phycobiont cells to develop its own symbiosis and grow further using the thallus host as substrate. Moya et al. (2020) analysed the microalgal diversity and interaction patterns in crustose lichens and lichenicolous lichens on gypsum by amplicon sequencing analysis of the nuclear internal transcribed spacer (nrITS) region and characterised the microalgae by ultrastructure analyses. They found that three microalgal genera formed the pool of potential phycobionts and were available for the lichenicolous lichens.

Diederich et al. (2018) reported a total of 257 species of lichenicolous lichens worldwide. It is likely that, in dry sites, the strategy of “stealing” the phycobiont is more widespread than currently assumed and that the 257 species listed by Diederich et al. (2018), as the 189 Italian species considered in this study, are the most specialised and evidently lichenicolous ones, just the “tip of the iceberg” of what could be the real lichenicolous lichens biota. Further research, using DNA amplicon sequencing and metagenomics, could lead to the discovery of new lichenicolous lichens species, from obligate to occasional, the latter stealing the phycobiont only in harsh environments.

## ﻿Conclusions

The results of the present study may be summarised as follows:

1. most lichenicolous lichens are crustose, with a non-trentepohlioid phycobiont;
2. they are clearly bound to sunny-dry habitats (rocks and soil);
3. such habitats seem to exert a positive selective pressure towards sexual reproduction of the mycobiont;
4. sexually reproducing species of dry habitats may encounter problems in the early stages of lichenisation and this has led to the evolution of “algal thieves”;
5. the number of “algal thieves” in dry habitats may be higher than currently assumed.

## ﻿Additional information

### Author ORCIDs

Pier Luigi Nimis https://orcid.org/0000-0003-3523-0183

Piero Pitacco https://orcid.org/0009-0001-0730-1362

Stefano Martellos https://orcid.org/0000-0001-5201-8948

Lucia Muggia https://orcid.org/0000-0003-0390-6169

### Data availability

All of the data that support the findings of this study are available in the main text.
